# Quality of routine health data at the onset of the COVID-19 pandemic in Ethiopia, Haiti, Laos, Nepal, and South Africa

**DOI:** 10.1186/s12963-023-00306-w

**Published:** 2023-05-20

**Authors:** Wondimu Ayele, Anna Gage, Neena R. Kapoor, Solomon Kassahun Gelaw, Dilipkumar Hensman, Anagaw Derseh Mebratie, Adiam Nega, Daisuke Asai, Gebeyaw Molla, Suresh Mehata, Londiwe Mthethwa, Nompumelelo Gloria Mfeka-Nkabinde, Jean Paul Joseph, Daniella Myriam Pierre, Roody Thermidor, Catherine Arsenault

**Affiliations:** 1 School of Public Health, College of Health Sciences, Addis Ababa University, Addis Ababa, Ethiopia; 2 Department of Global Health and Population, Harvard T.H. Chan School of Public Health, Boston, USA; 3 Ministry of Health of Ethiopia, Addis Ababa, Ethiopia; 4 World Health Organization, Vientiane, Lao People’s Democratic Republic; 5 Ethiopian Public Health Institute, Addis Ababa, Ethiopia; 6 Ministry of Health and Population, Government of Nepal, Kathmandu, Nepal; 7 School of Nursing and Public Health, University of KwaZulu-Natal, Durban, South Africa; 8 Division d’Épidémiologie et de Laboratoire, Zanmi Lasante, Mirebalais, Plateau Central Haiti; 9 Programme National de Lutte contre les IST/VIH/SIDA (PNLS) Unite de Coordination des Maladies Transmissibles (UCMIT), Ministère de la Sante Publique et de la Population (MSPP), Port-au-Prince, Haiti; 10 Studies and Planning Unit, Ministry of Public Health and Population, Port-au-Prince, Haiti

**Keywords:** Health management information systems, DHIS2, Data quality, COVID-19

## Abstract

**Background:**

During the COVID-19 pandemic, governments and researchers have used routine health data to estimate potential declines in the delivery and uptake of essential health services. This research relies on the data being high quality and, crucially, on the data quality not changing because of the pandemic. In this paper, we investigated those assumptions and assessed data quality before and during COVID-19.

**Methods:**

We obtained routine health data from the DHIS2 platforms in Ethiopia, Haiti, Lao People’s Democratic Republic, Nepal, and South Africa (KwaZulu-Natal province) for a range of 40 indicators on essential health services and institutional deaths. We extracted data over 24 months (January 2019–December 2020) including pre-pandemic data and the first 9 months of the pandemic. We assessed four dimensions of data quality: reporting completeness, presence of outliers, internal consistency, and external consistency.

**Results:**

We found high reporting completeness across countries and services and few declines in reporting at the onset of the pandemic. Positive outliers represented fewer than 1% of facility-month observations across services. Assessment of internal consistency across vaccine indicators found similar reporting of vaccines in all countries. Comparing cesarean section rates in the HMIS to those from population-representative surveys, we found high external consistency in all countries analyzed.

**Conclusions:**

While efforts remain to improve the quality of these data, our results show that several indicators in the HMIS can be reliably used to monitor service provision over time in these five countries.

**Supplementary Information:**

The online version contains supplementary material available at 10.1186/s12963-023-00306-w.

## Background

National health management information systems (HMIS) are important components of a health system, which provide data for policy formulation, planning, monitoring, and evaluation of health interventions, resource allocation, and day-to-day management decisions based on routine activities [1, 2]. The strengthening of health systems at the local, national, and international levels depends on reliable and timely health information. Over the past decade, national ministries of health, in collaboration with stakeholders, have made significant investments in strengthening routine health information systems in low- and middle-income countries [3–8].

The DHIS2 software, initially developed by the University of Oslo, is an open-source routine health data tracking platform that has now been adopted by more than 70 countries [9]. The purpose of DHIS2 is to facilitate reporting and improve health facilities’ ability to fulfill reporting requirements [10]. While there have been numerous efforts to improve routine health information systems and data quality, governance issues continue to hinder their ability to produce quality data and contribute to decision-making [3–9]. Data accuracy varies by health program area, including maternal and child health, infectious diseases, and non-communicable diseases [9].

Governments and researchers have been using routine health data to estimate interruptions in essential health services during the COVID-19 pandemic [11–14]. Conclusions about the resilience of health service provision during the pandemic and evidence on the magnitude of disruptions rely on the quality of routine health data [12]. Paper-based data collection and compilation at the primary source is subject to inconsistency, incompleteness and missing values, and data entry errors [15]. Other factors may have impaired data quality during the pandemic, including fears of handling paper, mobility restrictions, disruptions to energy or internet access, or lack of performance review or data quality assurance system. Poor data quality during the pandemic and a decline in reporting completeness could lead to over-reporting the magnitude of disruptions in health service use during the pandemic. Nevertheless, few analyses have been conducted to assess the quality of routine health data during the COVID-19 pandemic.

## Methods

In this analysis, we assessed the quality of routine health data extracted from DHIS2 platforms in five countries before the COVID-19 pandemic and during the initial months of the pandemic. We assess quality based on four dimensions drawn from the World Health Organization (WHO) Data Quality Review framework [16]: reporting completeness, presence of outliers, internal consistency, and external consistency.

### Data sources

We examined data on service utilization and institutional deaths extracted from the DHIS2 platforms in five countries: Ethiopia, Haiti, Lao People’s Democratic Republic (PDR), Nepal, and South Africa. These countries were included because they participated in a multi-country study on the impacts of COVID-19 on essential health services [11] and because they used the DHIS2 platform. In Haiti and Nepal, these data represented all potential health facilities in the country. In Ethiopia, the data excluded the Tigray Region due to the conflict in the region which began in the last months of 2020 and led to the closure of health facilities. In South Africa, the data were from the KwaZulu-Natal (KZN) province only. In Lao PDR, the data included only public sector facilities, which cover services provided to the majority of the population.

The data were extracted from DHIS2 at the lowest administrative level possible in each country. In Ethiopia, the dataset combined facility-level and district (*woreda*)-level data. Facility-level information was available for hospitals and large private facilities, while data from small facilities (health centers and health posts) were aggregated and reported by woreda health offices. In Nepal, data were obtained at the district (*palika*) level. In Haiti, Lao PDR and KwaZulu-Natal, the data were available at health facility levels.

We extracted routine monthly data on health services and institutional deaths for the period of January 1, 2019, to December 31, 2020, in South Africa, Laos, and Haiti. In Ethiopia, the data were obtained from Tahsas 2011 to Tahsas 2013 (equivalent to approximately December 30, 2018, to December 29, 2020). In Nepal, the data were obtained from Magh 2075 to Poush 2077 (equivalent to January 15, 2019, to January 13, 2021). For our analysis, the pre-pandemic period includes 15 months, from January 2019 to March 2020, and the pandemic period includes 9 months, from April to December 2020. In Nepal, the pre-pandemic period includes 14 months and the pandemic period 10 months.

We aimed to include health services for a wide range of conditions facing different age-groups, as well as commonly reported institutional deaths. We focused on indicators that were reported monthly for the purpose of our initial multi-country study which used an interrupted-time series design and required frequent data points.

### Data cleaning

We conducted minimal data cleaning before assessing data quality. First, in small facilities where few deaths occur, the reporting of institutional death indicators varied between 0 and missing. In some instances, we opted to impute values of 0 in order to assess data completeness. If a facility (or reporting unit) provided the health service related to the death indicator (i.e., inpatient admissions for inpatient deaths or deliveries for maternal, neonatal mortality, and stillbirths) any given month, missing values were imputed to be 0. If the facility did not report providing the service that month, the institutional mortality indicator was set to missing. Second, in Nepal and South Africa, there was further inconsistency for the cesarean section indicator which was sometimes reported as 0 or missing in facilities where c-sections were not provided. For all facilities where the sum of the c-section indicator across the total study period was 0 (or missing), we set all values to missing (the facility did not report the indicator). This resulted in excluding those facilities from the analysis for the c-section indicator.

### Analysis

We analyzed four aspects of data quality using the WHO Data Quality Review framework and metrics: completeness, outliers, and internal and external consistency [16].

For each indicator, we first assessed reporting completeness each month by dividing the number of units reporting each month by the expected number of units that should be reporting. Because updated master facility lists were not available, the expected number of facilities was based on the maximum number of units reporting during any given month over the two-year period. We assessed completeness during the pre-pandemic and pandemic periods separately and estimated the difference in completeness by period for each indicator. While there is no globally accepted threshold for adequate completeness, the WHO suggests benchmarks of 80% or 90% [16].

Second, we assessed the frequency of outliers over the study period. Outliers were defined as service or death counts in any given month that were greater than 3.5 standard deviations from the facility or administrative area mean over 24 months [16]. Low outliers (values lower than 3.5 standard deviations from the facility mean) were not assessed in the primary analysis as service counts may have legitimately decreased to low levels during the pandemic. In a secondary analysis, we separately assessed positive and negative outliers in the pre-pandemic and pandemic periods.

We next assessed the internal consistency of reporting between three related vaccine indicators in 2019 that should be given to infants within the first months of life: BCG, pentavalent, pneumococcal, oral poliovirus, and rotavirus vaccines. BCG is administered as soon as possible after birth. Pentavalent, pneumococcal, and oral polio are three dose courses typically given on the same days starting at 6 weeks after birth. Rotavirus is a two-dose course that is typically given with the first two doses of the other series. Because these vaccines are administered near the same time for each child, there should be similar numbers of vaccinations reported across the country. Haiti was excluded from this analysis due to the lack of vaccine-specific indicators in our dataset.

Finally, we assessed the external consistency of the pre-pandemic data (calendar year 2019) against a gold standard for one indicator: cesarean section rates. We first estimated the total absolute number of births taking place in the country or province (for KZN) in 2019 by dividing the total deliveries reported in HMIS by the rate of facility deliveries obtained from the most recent population-based survey (Demographic and Health Survey (DHS) or Multiple Indicator Cluster Survey (MICS)) [17, 18]. In Lao PDR, we used the public facility delivery rate from MICS because the HMIS only includes data from public sector facilities. In South Africa, we used estimates from the KZN province reported in the South African DHS 2016.

Second, we divided the absolute number of cesarean sections reported in HMIS by this estimated number of births in the country or province. We compared this estimate of population-based c-section rates to the one estimated in population-representative surveys. This method was based on the assumption that the HMIS contains all cesarean sections and facility deliveries performed in the country (or province), while the DHS and MICS provide accurate population-based estimates of facility delivery rates and cesarean section rates. We only considered the 2019 period prior to the pandemic so that any pandemic changes in facility delivery rates would not affect the estimate and because population-based surveys were all conducted before 2020.

The surveys used were the Ethiopia DHS 2019, South Africa DHS 2016, Lao PDR MICS 2017, and Nepal MICS 2019. Haiti was excluded because our dataset did not include c-sections. Given the older surveys, our estimate of the c-section rate in KZN and Lao PDR assumes that the population-based facility delivery rate remained constant between when the survey was conducted and 2019. Nonetheless, to account for possible changes in the facility delivery rates in KZN and Lao PDR, we estimated what the cesarean section rate would be if the facility delivery rate increased or decreased by 3 percentage points by 2019 to estimate an uncertainty interval.

## Results

Table 1 shows the list of indicators and number of facilities or administrative units reporting each indicator in each country. A total of 40 indicators were compiled including for reproductive, maternal, and neonatal health, child health, infectious diseases, non-communicable diseases (NCDs), and other health services. Detailed definitions for each indicator are available in Additional file 1.

**Table 1:** Maximum facilities or administrative units reporting each indicator over the study period

	Ethiopia	Haiti	South Africa	Nepal	Lao PDR
Level of reporting	Facilities and woredas	Facilities	Facilities	Palikas	Facilities
*RMNH services*					
Family planning	1900	617		753	1229
Antenatal care services	1640	708	1029	751	1161
Deliveries	1574	271	1029	700	920
Cesarean section	390		87	153	52
Neonatal resuscitation	613				
Kangaroo mother care	383				
Postnatal care visits	1571	380	867	661	1048
*Child health services*					
Acute malnutrition visits	1423		866	418	
Diarrhea visit	1099		65	752	
Pneumonia visit	1475		855	736	
BCG vaccines	1482		912	749	1123
Measles vaccine	1469		863	753	1008
Polio vaccine	1500			750	1171
Pentavalent vaccine	1502		863	753	1202
Pneumococcal vaccine	1501		864	752	1199
Rotavirus vaccine	1500		864		
Fully vaccinated by age 1	1422	456	863		
*Infectious disease services*					
HIV testing				211	
ART	1900		1029		
HIV suppression	650				
TB screening			948	528	
TB treatment			937		
NCD services					
Cervical cancer screening	393	34	1001		
Diabetes screening	864				
Diabetes treatment	537	405	940	537	201
Hypertension screening	1097				
Hypertension treatment	708	653		734	827
*Other services*					
Emergency room visits	917			177	
Inpatient visits	975		1029	103	1084
Trauma visits			69		
ICU visits			16		
Outpatient visits	1872	793	70	752	1229
Road traffic accidents	851		58		532
*Institutional deaths*					
Emergency room deaths	830				
ICU deaths	79		16		
Inpatient deaths	951		90	91	
Maternal deaths	1264	307	441	702	1038
Newborn deaths	1111		442	605	
Stillbirths	662	253	441	431	973
Trauma deaths			66		

The variation in the number of facilities or administrative units reporting a specific indicator within a country and between services is due to the availability of where services are provided. *RMNH* Reproductive, maternal, and newborn health; *NCD* Non-communicable diseases; *ICU* Intensive care unit

Although we aimed to obtain a comprehensive and comparable set of indicators across all countries, our compiled list does not cover the entire set of indicators available in countries. Indicators were missing either because they were not available on a monthly basis (for example, tuberculosis care in Ethiopia was excluded because it was only available quarterly) or because of various challenges with data extraction (e.g., lack of integration between various data systems in Haiti). In our dataset, Ethiopia and South Africa included the most indicators, while the fewest indicators were obtained in Haiti due to data extraction issues and challenges matching across a variety of data platforms. For example, HIV-related indicators are managed on a different data system in Haiti [19].

Across all countries, we were unable to obtain any indicators related to mental health care or surgical care (except for cesarean sections). Ethiopia was the only country that obtained indicators related to the quality of health services (e.g., number of newborns resuscitated who survived, number of diabetic patients with a controlled condition).

**Fig. 1:**
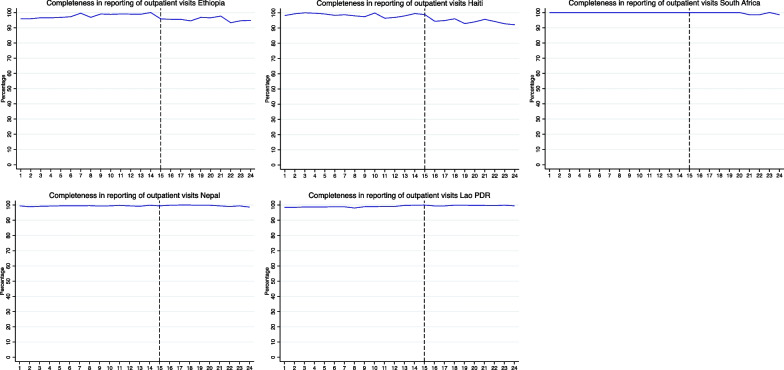
Completeness in reporting of total outpatient visits over time January 2019 to December 2020 in five countries. Months 1 to 24 are January 2019 to December 2020. The vertical line at month 15 represent April 2020 and the beginning of the COVID-19 pandemic. Monthly reporting completeness for all other indicators are available on a GitHub repository: GitHub repository: https://github.com/catherine-arsenault/HS-performance-during-covid-do-files/tree/master/Multi-country/Paper%202%20Data%20quality

Completeness findings are shown in Table 2 and Fig. 1. Reporting completeness was greater than 95% across most indicators and countries, surpassing the WHO-suggested benchmarks. Nonetheless, lower reporting completeness persists in some countries, particularly for NCD services, infectious disease services, and ICU visits and deaths. Ethiopia substantially increased the reporting of NCD services over the study period, as these indicators were added to the DHIS2 system in late 2019 and the registers were progressively distributed to facilities. Ethiopia and Nepal may have higher completeness because reporting is at an administrative level rather than individual facilities.

**Table 2:**
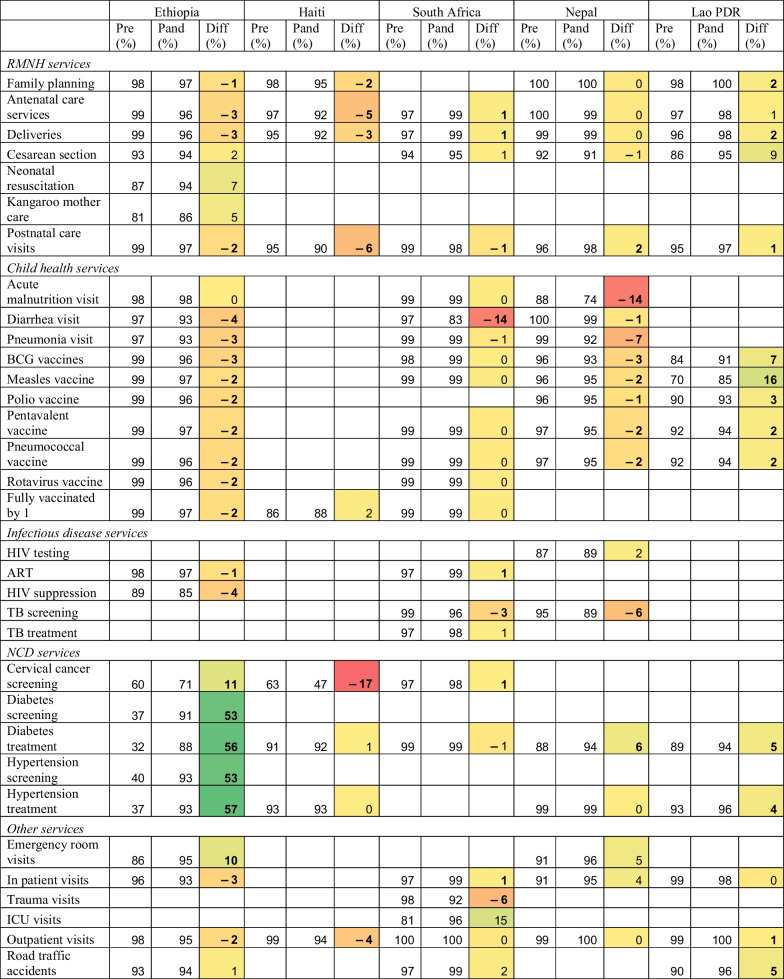

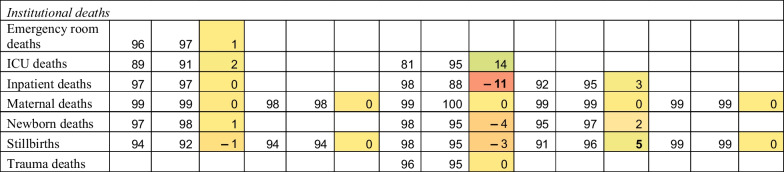
Completeness of reporting in the pre-pandemic and pandemic periods in five countries

Bolded differences indicate statistically significant changes from pre-pandemic to pandemic period (*p*-value for two tailed *t* test < 0.05). Colors show size of difference. *Pre* Pre-pandemic period; *Pand* Pandemic period; *Diff* Difference; *RMNH* Reproductive, maternal and newborn health; *NCD* Non-communicable diseases; *ICU* Intensive care unit

For most indicators and countries, we found no evidence that the onset of the COVID-19 pandemic substantially affected reporting completeness. On average, reporting increased by 2 percentage points across the study indicators and countries. This could be due to improvements in reporting in some countries where the systems are relatively new and data quality has been steadily improving. The largest declines in reporting were seen in Haiti for cervical cancer screening (− 17%), in South Africa for diarrhea (− 14%) and inpatient deaths (− 11%), and in Nepal for acute malnutrition visits (− 14%). Because these declines in reporting are only seen for particular services across the study countries, it is unlikely that entire facilities stopped reporting during the pandemic. However, if service coverage declined so precipitously such that facilities were no longer providing a given service, it may be considered incomplete. Further qualitative research is needed to understand why reporting declined for these particular services during the pandemic. Figure 1 shows reporting completeness for outpatient visits from January 2019 to December 2020. A slight decline in completeness may be seen in Ethiopia and Haiti, but remained above 90% each month pre- and post-COVID-19.

Positive outliers were infrequent across study countries and indicators (Table 3). A maximum of 0.59% of facility-months had values greater than 3.5 standard deviations from the average for the facility, which occurred among inpatient deaths in KZN. For this indicator, the positive outliers were higher in the pre-COVID-19 period (1.4%) in comparison with the pandemic period (0.1%) (Additional file 1). No indicators in any country had a positive outlier frequency above 1%. Vaccination services were slightly more likely to have positive outliers than other services, but the frequency was still below 0.5% for all indicators in all countries.

**Table 3:** Frequency of positive outlier over the study period

	Ethiopia (%)	Haiti (%)	South Africa (KZN) (%)	Nepal (%)	Lao PDR (%)
*RMNH services*					
Family planning	0.32	0.42		0.16	0.22
Antenatal care	0.19	0.48	0.08	0.23	0.21
Deliveries	0.08	0.11	0.02	0.08	0.08
Cesarean sections	0.07		0.05	0.03	0.00
Neonatal resuscitation	0.21				
Kangaroo mother care	0.05		0.69		
Postnatal care	0.18	0.19	0.05	0.16	0.18
*Child health services*					
Acute malnutrition visits	0.45		0.02	0.19	
Diarrhea visits	0.42		0.13	0.19	
Pneumonia visits	0.25		0.36	0.25	
BCG vaccine	0.21		0.48	0.09	0.23
Measles vaccine	0.38		0.13	0.04	0.28
Polio vaccine	0.27			0.05	0.09
Pentavalent vaccine	0.25		0.10	0.03	0.10
Pneumococcal vaccine	0.24		0.11	0.26	0.11
Rotavirus vaccine	0.18		0.10		
Fully vaccinated by 1	0.37	0.35	0.10		
*Infectious disease services*					
HIV testing				0.31	
ART	0.21		0.04		
HIV suppression	0.22				
TB screening			0.06	0.18	
TB treatment			0.06		
*NCD services*					
Cervical cancer screening	0.41	0.50	0.57		
Diabetes detection	0.15				
Diabetes visits	0.04	0.49	0.43	0.22	0.16
Hypertension detection	0.09				
Hypertension visits	0.02	0.35		0.23	0.15
*Other services*					
Emergency room	0.21			0.51	
Intensive care unit	0.06		0.00		
Inpatient visits	0.45		0.00	0.22	0.28
Outpatient visits	0.26	0.39	0.00	0.18	0.27
Road traffic accidents	0.46		0.15		0.13
Trauma visits			0.25		
*Institutional deaths*					
Emergency room deaths	0.13				
ICU deaths			0.00		
Inpatient deaths	0.12		0.59	0.25	
Maternal deaths	0.00	0.00	0.00	0.00	0.00
Newborn deaths	0.12		0.00	0.04	0.00
Stillbirths	0.16	0.03	0.02	0.08	
Trauma deaths			0.07		0.24

*RMNH* Reproductive, maternal and newborn health; *NCD* Non-communicable diseases; *ICU* Intensive care unit

Negative outliers were more common than positive outliers, with the highest rates seen in Haiti for cervical cancer screening (5.3%). However, most indicators in most countries had negative outlier frequency below 1% as well. These rates remained constant across the pre-pandemic and pandemic periods, suggesting outliers did not increase in the initial months of the pandemic (Additional file 1).

Regarding internal consistency, compared to the three-dose pentavalent vaccine, the number of other vaccines provided ranged from 91 to 110% of pentavalent (Table 4). The largest differences in reporting were for BCG. Differences between BCG (given at birth) and the other vaccines delivered later in the infant’s life could be explained by the fact that there is a drop off after BCG or because children born outside of health facilities do not receive BCG or BCG is recorded elsewhere. Across the other four vaccines, the smallest differences were observed in Ethiopia (less than 2% difference between all vaccines) and the largest in South Africa (12% difference between pneumococcal and rotavirus vaccines).

**Table 4:** Internal consistency between related vaccine service indicators in 2019

	3rd dose Pentavalent	BCG		3rd dose Pneumococcal		3rd dose Oral Polio		2nd dose Rotavirus	
		Number	% Penta3 (%)	Number	% Penta3 (%)	Number	% Penta3 (%)	Number	% Penta3 (%)
Ethiopia	5,962,794	6,070,807	102	5,922,534	99	5,877,936	99	5,886,793	99
South Africa	421,784	385,619	91	464,272	110			413,308	98
Lao PDR	270,512	263,755	98	266,734	99	253,542	94		
Nepal	1,026,692	1,102,239	107	982,775	96	1,000,842	97		

% Penta3 is the number of BCG, pneumococcal, oral polio and rotavirus vaccines divided by the number of 3rd dose pentavalent vaccines

**Fig. 2:**
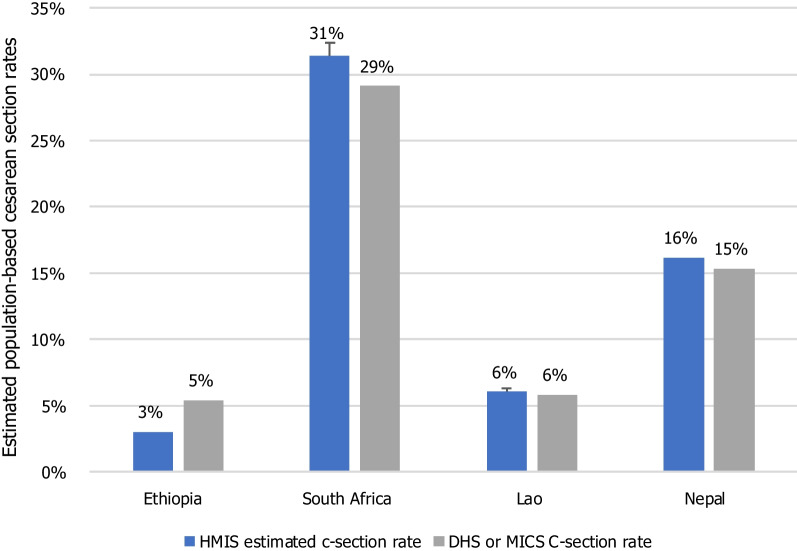
External consistency of cesarean section rate in the 2019 HMIS in comparison with the most recent DHS or MICS surveys in four countries. Notes: Survey data are the Ethiopia DHS (2019), South Africa DHS (2016), Lao MICS (2017), and Nepal MICS (2019)

All countries had good external consistency for the HMIS-based c-section rate (Fig. 2). Lao PDR and Nepal had the highest external consistency of cesarean section rates, with nearly identical rates between the HMIS and the MICS. In Nepal, this may be because reporting cesarean sections in HMIS is mandatory for both public and private facilities in order to receive reimbursement from a social security program. In Ethiopia, there appeared to be a slight underreporting of cesarean sections in the HMIS system compared with the DHS survey (− 2 percentage points (two-tailed *t* test of proportions *p* < 0.01)). This may be due to underreporting of private facilities to the HMIS, where many cesarean sections occur or to incomplete reporting in public facilities [20]. Another analysis comparing the number of cesarean sections reported in the Ethiopian HMIS to those found in operating theater paper-based registers also found some underreporting of c-sections in the HMIS [21]. In South Africa, the HMIS rate was 2 percentage points higher than the DHS rate. The difference may simply be due to the fact that the DHS was conducted 3 years prior.

## Discussion

Our analysis of the quality of routine health data in five countries found that reporting completeness was high for most indicators and did not decrease significantly during the initial months of the pandemic. However, low reporting completeness is persistent in some countries, but the extent varies according to different indicators. There were more negative outliers than positive ones, but their frequency was less than 1% for most countries. The internal consistency among vaccination indicators was generally high. An examination of external consistency revealed high consistency of c-section rates between the HMIS and population-based surveys. The completeness and presence of outliers did not show a significant difference before and during the COVID-19 pandemic.

Stable completeness and limited impacts on data quality during COVID were also observed in several other studies [14, 22]. A study of routine data from twelve sub-Saharan African countries found completeness over 90% in most countries and no major declines during 2020 [14]. A second study of eighteen low- and middle-income countries found varied completeness by country and indicator, with Nigeria experiencing significant disruption in reporting during the initial period of the pandemic while other countries remained more stable [22]. These studies did not examine the presence of outliers, internal consistency, and external consistency in the context of the pandemic.

Other challenges outside of data quality continue to impede the widespread use of HMIS for research and policy. First, in some countries it is difficult to find a continually updated and publicly available master facility list. The total number of facilities that should be reporting each indicator through the HMIS is thus often unclear. There are also potential challenges with representativeness. For example, in Lao PDR and several other countries not included in this analysis the HMIS system only includes public sector facilities. In Lao PDR, the Ministry of Health has initiated a process of including larger private facilities in the capital city to contribute regularly in the HMIS. Third, there are limitations in the types of indicators that are reported in the HMIS. For example, data on non-communicable diseases, which are increasing as countries undergo the epidemiologic transition, were limited particularly in South Africa, Nepal, and Lao PDR. We found no information on mental health care or surgical services in any country besides c-sections. Most of the indicators provided were also related to the volume of services provided, with limited information on the quality of care (processes and outcomes). Data on quality of care would be helpful to track the performance of health systems. For example, the proportion of diabetic or hypertensive patients with controlled conditions, or the number of surgical site infections, would allow monitoring health system quality through the DHIS2. Nonetheless, data aggregated at the facility level is often not conducive to examining quality of care which must often be measured at the patient level. As routine health data systems continue to improve, countries should consider integrating patient-level electronic health records into their HMIS (for example, through the “tracker” feature in DHIS2), in order to integrate monitoring of quality of care and facilitate more comprehensive data collection and management [23]. Finally, some countries need to improve integration between various health data monitoring platforms. For example, in Haiti the HIV database could be integrated into the DHIS2 platform, and paper-based and electronic-based systems need to be integrated.

Our analysis of data quality has several limitations to note. First, the indicators available from the HMIS in each country are more extensive than what the research team was able to obtain. Second, there are multiple ways of measuring completeness of an indicator using different denominators of facilities that may provide a given service. For example, in the most conservative method, the denominator would be the total number of facilities that ever-reported a given indicator at least once. In our method, the denominator was the maximum number of facilities that reported an indicator from any month across the study period. Third, in Ethiopia, Haiti, and Lao PDR, there was no distinction between a value of “0” and missing for most indicators. Because we did not impute zeros in these cases for health service volumes (only for deaths as discussed above), completeness rates are lower than they would be if zeros were entered separately from missing. Fourth, the external consistency assessment is limited to one indicator with gold standard population measurement, and our analysis relies on assumptions for calculating total births. Fifth, we only include the first nine months of the COVID-19 pandemic (April to December 2020), when countries did not face high COVID caseloads. In Lao PDR, for example, only 41 total COVID cases had been reported by December 31, 2020. As countries often faced larger waves of COVID in 2021 and 2022, future analyses must assess changes in data quality in these years. Finally, we do not assess the timeliness of reporting nor the consistency of population data used to calculate intervention coverage measures, two dimensions also included in the WHO Data Quality Review [16], though individual countries, including Lao PDR, may assess timeliness regularly.

## Conclusions

Efforts remain to improve the quality and accuracy of routine health data in many low- and middle-income countries. Priorities include improving reporting completeness, aligning the types of indicators reported with the disease burden, improving reporting by private sector health facilities, and improving feedback mechanisms to health facilities and the lowest administrative reporting levels. Countries must also ensure that the types of indicators reported are useful to health system planning and monitoring of quality of care and health outcomes. Nonetheless, our results show that a majority of indicators we collected can be used to monitor service provision over time. As the data quality improves, it will be important to expand the use of these locally owned routine data systems in research and policy decisions. Ultimately, HMIS should support decision-making and help identify priorities for health system improvements.

## Supplementary Information

**Additional file 1: Table 1.** Indicator definitions. **Table 2.** Positive and negative outliers in the pre-pandemic and pandemic periods (Ethiopia and Haiti). **Table 3.** Positive and negative outliers in the pre-pandemic and pandemic periods (South Africa and Nepal). **Table 4.** Positive and negative outliers in the pre-pandemic and pandemic periods (Lao PDR).

## Data Availability

The data that support the findings of this study are available from the countries’ respective Ministries of Health, but restrictions apply to the availability of these data, which were used under agreement for the current study, and so are not publicly available.

## Abbreviations

BCG: Bacille Calmette–Guerin
DHIS2: District health information system 2
DHS: Demographic and Health Surveys
HIV: Human immunodeficiency virus
HMIS: Health management information systems
ICU: Intensive care unit
KZN: KwaZulu-Natal
MICS: Multiple Indicator Cluster Survey
NCD: Non-communicable diseases
PDR: People’s Democratic Republic
WHO: World Health Organization
COVID-19: Coronavirus disease 2019
