# Supplementary material for: Quality of routine health data at the onset of the COVID-19 pandemic in Ethiopia, Haiti, Laos, Nepal, and South Africa
Source: Popul Health Metr. 2023 May 20;21:7. doi: 10.1186/s12963-023-00306-w (PMC10199286; doi:10.1186/s12963-023-00306-w)
Supplement: Supplementary file 1 — Additional file 1: Table 1. Indicator definitions. Table 2. Positive and negative outliers in the pre-pandemic and pandemic periods (Ethiopia and Haiti). Table 3. Positive and negative outliers in the pre-pandemic and pandemic periods (South Africa and Nepal). Table 4. Positive and negative outliers in the pre-pandemic and pandemic periods (Lao PDR). [file 12963_2023_306_MOESM1_ESM.docx]

**Quality of routine health data at the onset of the COVID-19 pandemic in Ethiopia, Haiti, Laos, Nepal, and South Africa**

**Supplementary materials**

**Supplementary Table 1. Indicator definitions**

|  |  | **Ethiopia** | **Haiti** | **Lao PDR** | **Nepal** | **South Africa (KZN** |
| --- | --- | --- | --- | --- | --- | --- |
| **Reproductive and maternal** | **Family planning** | Total new and repeat acceptors disaggregated by method | Visites des Clientes PF \| Repartition des vistes | Combined pill New user Combined pill Continue user  Emergency pill New user  Emergency pill Continue user  Single pill new users  Single pill Continue users Depose (Injectable) New users  Depose (Injectable) Continue users  Condoms New users  Condoms Continue users | Family Planning Program - Temporary FP Method - Depo-Current User +  Family Planning Program - Temporary FP Method - Depo-New Users < 20 Years +  Family Planning Program - Temporary FP Method - Depo-New Users > 20 Years +  Family Planning Program - Temporary FP Method - Pills- Current User +  Family Planning Program - Temporary FP Method - Pills- < 20 Years +  Family Planning Program - Temporary FP Method - Pills- > 20 Years +  Safe Motherhood Program-Safe Abortion Service-Post Abortion FP Methods Short Term-Medical +  Safe Motherhood Program-Safe Abortion Service-Post Abortion FP Methods Short term-Surgical + |  |
|  | **Antenatal care** | Number of pregnant women that received ANC at least once by maternal age | Visites des Femmes Enceintes \| Repartition des visites | ANC 1st visit by Health facility/Outreach | Safe Motherhood Program-Antenatal Checkup-First ANC visits (any time) < 20 years + Safe Motherhood Program-Antenatal Checkup-First ANC visits (any time) > 20 years | Number of first antenatal care visits total |
|  | **Deliveries** | Total number of births attended by skilled health personnel | Accouchements Institutionnels | Delivery at HF | Safe Motherhood Program-Delivery Service-Skilled Birth Attendants (SBA)Facility | Delivery in facility sum |
|  | **Caesarean sections** | Number of women having given birth by caesarean section |  | Caesarean delivery | Safe Motherhood Program- Type of Delivery - C/S Breech + Safe Motherhood Program- Type of Delivery - C/S Cephalic +  Safe Motherhood Program- Type of Delivery - C/S Shoulder | Delivery by caesarean section |
|  | **Neonatal resuscitation** | Total number of neonates resuscitated and survived |  |  |  |  |
|  | **Kangaroo mother care** | Total number of newborns weighting <2000gm and/or premature newborns for which KMC initiated |  |  |  |  |
|  | **Postnatal care** | Number of postnatal visits within 7 days of delivery | Consultations postnatales | PNC within 2 days + PNC 3-42 days | Safe Motherhood Program- 3 PNC visits as per protocol | Infant postnatal visit within 6 days after delivery |
| **Child health** | **Diarrhea** | Number of children treated for diarrhea with ORS only |  |  | CBIMCI-(2-59Months)- Classification-Diarrhea-Dysentery + CBIMCI-(2-59Months)- Classification-Diarrhea-No Dehydration +  CBIMCI-(2-59Months)- Classification-Diarrhea-Prolonged Diarrhea + CBIMCI-(2-59Months)- Classification-Diarrhea-Severe Dehydration +  CBIMCI-(2-59Months)- Classification-Diarrhea-Some Dehydration | Child under 5 years with diarrhoea as main final diagnosis at separation |
|  | **Malnutrition** | Total number of children <5yrs screened for acute malnutrition |  |  | CBIMCI-(2-59Months)- Classification-Severe Malnutrition | Severe acute malnutrition in child under 5 years new |
|  | **Pneumonia** | Number of children under 5 treated for pneumonia |  |  | CBIMCI-(2-59Months)- Classification-ARI-Pneumonia +  CBIMCI-(2-59Months)-ORC Classification-ARI-Severe Pneumonia/Very Severe Disease | Child under 5 years classified as pneumonia according to the IMCI definition |
| **Child vaccinations** | **BCG** | Number of children under one year of age who have received BCG vaccine |  | BCG | Immunization program - Vaccine Type- Children Immunized - BCG Doses | BCG (tuberculosis) vaccine given to a child under one year of age at birth. |
|  | **Pentavalent** | Number of children under one year who have received third dose of pentavalent vaccine |  | Penta 3 | Immunization program - Vaccine Type- Children Immunized - DPT-HepB-Hib 3rd | DTaP-IPV Hib-HBV (Hexavalent) 3rd dose given to a child under one year |
|  | **Polio** | Number of children under one year of age who have received third dose of Polio vaccine |  | Children got IPV 1 | Immunization program - Vaccine Type - Doses Children Immunized - OPV-3rd |  |
|  | **Pneumococcal** | Number of children under one year of age who have received third dose of pneumococcal vaccine |  | PCV 3 | Immunization program - Vaccine Type - Doses Children Immunized - PCV-3rd | Pneumococcal (PCV) vaccine 3rd dose given to a child under one year |
|  | **Rotavirus** | Number of children under one year of age who have received 2nd dose of Rotavirus vaccine |  |  |  | Rota Virus (RV) vaccine 2nd dose given to a child under one year |
|  | **Measles** | Number of children under one year who received first dose of measles vaccine |  |  | Immunization program - Children Immunized - Measles/Rubella - 9-11 Months + Immunization program - Children Immunized - Measles/Rubella - 12-23 Months | Measles vaccine 2nd dose given to a child at 12 months after birth |
|  | **Fully vaccinated by 1** | Number of children received all vaccine doses before 1st birthday | Complètement Vaccinés Communautaires + Completement Vaccinés Institutionnelles |  |  | Immunized fully under one year new |
| **Infectious disease** | **People on ART** | Number of adults and children who are currently on ART |  |  |  | Total clients remaining on ART (TROA) end of month - sum |
|  | **HIV tests** |  |  |  | Virology-HIV tests conducted |  |
|  | **HIV suppression** | Number of adult and pediatric patients with undetectable viral load <1,000 copies/ml in reporting period |  |  |  |  |
|  | **TB screening** |  |  |  | Disaggregation by Sex & Caste/Ethnicity- New TB Cases | Clients 5 years and older who were screened in health facilities for TB symptoms using the standard TB screening tool as per National TB Guideline |
|  | **TB treatment** |  |  |  |  | Clients 5 years and older who were started on TB treatment regimen |
|  | **Diabetes screening** | Total number of individuals screened for diabetes in the reporting period |  |  |  |  |
| **Non communicable diseases** | **Diabetes treatment** | Total number of diabetic patients enrolled to care six month prior to the report | Anciens Cas Diabete+ Nouveaux Cas Diabete | OPD Diabetes | Outpatient Morbidity-Nutritional & Metabolic Disorder-Diabetes Mellitus (DM) Cases | Diabetes treatment visit (Every visit for routine care by clients 40 years and older on treatment for diabetes) |
|  | **Hypertension screening** | Total number of individuals screened for diabetes in the reporting period |  |  |  |  |
|  | **Hypertension treatment** | Total number of hypertensive patients enrolled to care six month prior to the report | Anciens Cas HTA + Nouveaux Cas HTA | OPD Hypertension | OPD-Morbidity-Cardiovascular & Respiratory Related Problems-Hypertension |  |
|  | **Cervical cancer screening** | Number of women aged 30-49 screened with VIA for cervical cancer | Nbre de Femmes benificiaires d'une inspection visuelle a l'acide acetique (IVAA) |  |  | Cervical cancer screening (pap smear, visual inspection with Acetic acid (VIA) and liquid base cytology) for women 30 years and older |
|  | **Road traffic accidents** | Number of road traffic injury cases disaggregated by accident type |  | OPD Road traffic injury + IPD Road traffic injury |  | Emergency case-Motor Vehicle Accident - Occupant + Emergency case - Motor Vehicle Accident - Pedestrian |
| **Other services** | **Outpatient visits** | Number of outpatient visits | Visites des Clientes PF \| Repartition des vistes + Visites des Enfants 1 - 4 ans \| Repartition des visites + Visites des Enfants 10 - 14 ans \| Repartition des visites + Visites des Enfants 5 - 9 ans \| Repartition des visites + Visites des Enfants < 1 an \| Repartition des visites + Visites des Femmes Enceintes \| Repartition des visites + Visites des Jeunes adultes 15 - 19 ans \| Repartition des visites + Visites des Personnes a mobilite reduite (moteur) + Visites des Personnes a mobilite reduite (sensoriel) + Visites des jeunes adultes 20 - 24 ans \| Repartition des visites + Vistes des Autres Adultes \| Repartition des visites | OPD: Outpatient visits | Disaggregation by Sex & Caste/Ethnicity - Outpatient Cases | Total clients attending general or specialist Outpatient clinics |
|  | **Emergency room visits** | Total number of emergency unit attendances |  |  | Clients Received Emergency Services - Age Group 0-9 Years + Clients Received Emergency Services - Age Group 10-19 Years + Clients Received Emergency Services - Age Group 20-59 Years +  Clients Received Emergency Services - Age Group >-60 Years |  |
|  | **Inpatient visits** | Number of inpatient admissions |  | IPD: Inpatient visits | Inpatient Morbidity Cases | Admissions - Total |
|  | **Trauma visits** |  |  |  |  |  |
|  | **ICU visits** |  |  |  |  | Admissions - trauma |
| **Deaths** | **Emergency room deaths** | Total death in emergency unit |  |  |  |  |
|  | **ICU deaths** | Total death in ICU in the reporting period |  |  |  | Inpatient death - ICU |
|  | **In patient deaths** | Number of inpatient deaths in the reporting period +  Total death in ICU in the reporting period |  |  | Inpatient morbidity Deaths | Inpatient deaths - total |
|  | **Maternal deaths** | Number of maternal deaths in health facility | Nombre de Décès maternels | Maternal deaths | Safe Motherhood Program - Maternal Death - Antepartum +  Safe Motherhood Program - Maternal Death - Intrapartum +  Safe Motherhood Program - Maternal Death - Postpartum | Maternal death in facility |
|  | **Newborn deaths** | Number of neonatal deaths in the first 7 days of life(Institutional) |  | Death Neonatal 0-7 days | Total Late Neonatal Deaths in the Hospital +  Safe Motherhood Program-Maternal Death-Neonatal death at Health Facility +  Total Early Neonatal Deaths in the Hospital | Death in facility 0-6 days |
|  | **Stillbirths** | Number of still births | Nombres de morts nés | Still birth >=28weeks | Safe Motherhood Program - Number of Still Births - Fresh +  Safe Motherhood Program - Number of Still Births - Macerated | Still birth in facility |
|  | **Trauma deaths** |  |  |  |  | Inpatient death - Trauma |

**Supplementary Table 2. Positive and negative outliers in the pre-pandemic and pandemic periods (Ethiopia and Haiti)**

|  | Ethiopia | | | | Haiti | | | |
| --- | --- | --- | --- | --- | --- | --- | --- | --- |
|  | Positive pre | Negative pre | Positive pandemic | Negative pandemic | Positive pre | Negative pre | Positive pandemic | Negative pandemic |
| RMNH services |  |  |  |  |  |  |  |  |
| Family planning | 0.4% | 0.1% | 0.3% | 0.1% | 0.2% | 0.2% | 0.6% | 0.3% |
| Antenatal care services | 0.3% | 0.1% | 0.2% | 0.1% | 0.3% | 0.1% | 0.6% | 0.0% |
| Deliveries | 0.1% | 0.1% | 0.1% | 0.1% | 0.1% | 0.4% | 0.1% | 0.3% |
| Cesarean section | 0.1% | 0.5% | 0.1% | 1.2% |  |  |  |  |
| Neonatal resuscitation | 0.3% | 0.4% | 0.3% | 0.6% |  |  |  |  |
| Kangaroo mother care | 0.0% | 0.8% | 0.1% | 1.0% |  |  |  |  |
| Postnatal care visits | 0.2% | 0.0% | 0.1% | 0.1% | 0.1% | 0.2% | 0.2% | 0.2% |
| Child health services |  |  |  |  |  |  |  |  |
| Acute malnutrition visit | 0.7% | 0.1% | 0.3% | 0.1% |  |  |  |  |
| Diarrhea visit | 0.3% | 0.2% | 0.5% | 0.3% |  |  |  |  |
| Pnuemonia visit | 0.2% | 0.1% | 0.3% | 0.1% |  |  |  |  |
| BCG vaccines | 0.3% | 0.1% | 0.2% | 0.1% |  |  |  |  |
| Measles vaccine | 0.5% | 0.2% | 0.3% | 0.1% |  |  |  |  |
| Polio vaccine | 0.4% | 0.1% | 0.2% | 0.1% |  |  |  |  |
| Pentavalent vaccine | 0.4% | 0.1% | 0.2% | 0.1% |  |  |  |  |
| Pneumococcal vaccine | 0.4% | 0.1% | 0.1% | 0.1% |  |  |  |  |
| Rotavirus vaccine | 0.3% | 0.1% | 0.1% | 0.1% |  |  |  |  |
| Fully vaccinated 1 year old | 0.5% | 0.1% | 0.3% | 0.1% | 0.4% | 0.4% | 0.3% | 0.4% |
| Infectious disease services |  |  |  |  |  |  |  |  |
| HIV testing |  |  |  |  |  |  |  |  |
| ART | 0.2% | 0.4% | 0.2% | 0.5% |  |  |  |  |
| HIV suppression | 0.2% | 0.5% | 0.2% | 0.5% |  |  |  |  |
| TB screening |  |  |  |  |  |  |  |  |
| TB treatment |  |  |  |  |  |  |  |  |
| NCD services |  |  |  |  |  |  |  |  |
| Cervical cancer screening | 0.6% | 3.5% | 0.3% | 2.1% | 0.0% | 5.2% | 0.8% | 5.5% |
| Diabetes screening | 0.2% | 0.9% | 0.1% | 0.8% |  |  |  |  |
| Diabetes treatment | 0.0% | 1.6% | 0.0% | 2.7% | 0.4% | 0.5% | 0.5% | 0.3% |
| Hypertension screening | 0.1% | 0.5% | 0.0% | 0.5% |  |  |  |  |
| Hypertension treatment | 0.0% | 1.4% | 0.0% | 1.3% | 0.2% | 0.1% | 0.4% | 0.1% |
| Other services |  |  |  |  |  |  |  |  |
| Emergency room visits | 0.2% | 0.6% | 0.2% | 0.5% |  |  |  |  |
| In patient visits | 0.6% | 0.4% | 0.4% | 0.3% |  |  |  |  |
| Trauma visits |  |  |  |  |  |  |  |  |
| ICU visits |  |  |  |  |  |  |  |  |
| Outpatient visits | 0.3% | 0.1% | 0.2% | 0.2% | 0.2% | 0.0% | 0.5% | 0.1% |
| Road traffic accidents | 0.4% | 0.5% | 0.5% | 0.5% |  |  |  |  |
| Deaths |  |  |  |  |  |  |  |  |
| Emergency room deaths | 0.1% | 0.6% | 0.0% | 0.7% |  |  |  |  |
| ICU deaths | 0.0% | 0.9% | 0.1% | 0.8% |  |  |  |  |
| In patient deaths | 0.0% | 0.3% | 0.1% | 0.4% |  |  |  |  |
| Maternal deaths | 0.0% | 0.1% | 0.0% | 0.1% | 0.0% | 0.1% | 0.0% | 0.2% |
| Newborn deaths | 0.0% | 0.2% | 0.0% | 0.3% |  |  |  |  |
| Stillbirths | 0.1% | 0.2% | 0.2% | 0.2% | 0.0% | 0.3% | 0.1% | 0.1% |
| Trauma deaths |  |  |  |  |  |  |  |  |

**Supplementary Table 3. Positive and negative outliers in the pre-pandemic and pandemic periods (South Africa and Nepal)**

|  | South Africa (KZN) | | | | Nepal | | | |
| --- | --- | --- | --- | --- | --- | --- | --- | --- |
|  | Positive pre | Negative pre | Positive pandemic | Negative pandemic | Positive pre | Negative pre | Positive pandemic | Negative pandemic |
| RMNH services |  |  |  |  |  |  |  |  |
| Family planning |  |  |  |  | 0.2% | 0.1% | 0.2% | 0.0% |
| Antenatal care services | 0.1% | 0.0% | 0.1% | 0.0% | 0.4% | 0.0% | 0.1% | 0.0% |
| Deliveries | 0.0% | 0.0% | 0.0% | 0.0% | 0.1% | 0.0% | 0.1% | 0.0% |
| Cesarean section | 0.1% | 0.1% | 0.0% | 0.0% | 0.1% | 0.5% | 0.0% | 0.6% |
| Neonatal resuscitation |  |  |  |  |  |  |  |  |
| Kangaroo mother care |  |  |  |  |  |  |  |  |
| Postnatal care visits | 0.1% | 0.0% | 0.0% | 0.0% | 0.2% | 0.0% | 0.1% | 0.0% |
| Child health services |  |  |  |  |  |  |  |  |
| Acute malnutrition visit | 0.0% | 0.0% | 0.0% | 0.0% | 0.1% | 0.1% | 0.2% | 0.2% |
| Diarrhea visit | 0.0% | 0.0% | 0.2% | 0.0% | 0.1% | 0.0% | 0.3% | 0.0% |
| Pnuemonia visit | 0.1% | 0.0% | 0.6% | 0.0% | 0.0% | 0.0% | 0.4% | 0.0% |
| BCG vaccines | 0.2% | 0.0% | 0.7% | 0.0% | 0.1% | 0.0% | 0.1% | 0.0% |
| Measles vaccine | 0.1% | 0.0% | 0.2% | 0.0% | 1.1% | 0.0% | 0.0% | 0.1% |
| Polio vaccine |  |  |  |  | 0.0% | 0.1% | 0.0% | 0.0% |
| Pentavalent vaccine | 0.1% | 0.0% | 0.1% | 0.0% | 0.0% | 0.1% | 0.0% | 0.0% |
| Pneumococcal vaccine | 0.1% | 0.1% | 0.1% | 0.0% | 0.7% | 0.0% | 0.0% | 0.0% |
| Rotavirus vaccine | 0.1% | 0.0% | 0.1% | 0.0% |  |  |  |  |
| Fully vaccinated 1 year old | 0.1% | 0.0% | 0.1% | 0.0% |  |  |  |  |
| Infectious disease services |  |  |  |  |  |  |  |  |
| HIV testing |  |  |  |  | 0.2% | 0.1% | 0.1% | 0.2% |
| ART | 0.0% | 0.2% | 0.0% | 0.1% |  |  |  |  |
| HIV suppression |  |  |  |  |  |  |  |  |
| TB screening | 0.0% | 0.1% | 0.1% | 0.1% |  |  |  |  |
| TB treatment | 0.0% | 0.0% | 0.1% | 0.0% |  |  |  |  |
| NCD services |  |  |  |  |  |  |  |  |
| Cervical cancer screening | 0.3% | 0.0% | 0.8% | 0.0% |  |  |  |  |
| Diabetes screening |  |  |  |  |  |  |  |  |
| Diabetes treatment | 0.2% | 0.0% | 0.6% | 0.0% | 0.3% | 0.1% | 0.2% | 0.1% |
| Hypertension screening |  |  |  |  |  |  |  |  |
| Hypertension treatment |  |  |  |  | 0.3% | 0.0% | 0.2% | 0.0% |
| Other services |  |  |  |  |  |  |  |  |
| Emergency room visits |  |  |  |  | 0.7% | 0.0% | 0.4% | 0.0% |
| In patient visits | 0.0% | 0.0% | 0.0% | 0.0% | 0.0% | 0.7% | 0.4% | 0.3% |
| Trauma visits | 0.3% | 0.0% | 0.2% | 0.0% |  |  |  |  |
| ICU visits | 0.0% | 0.0% | 0.0% | 0.0% |  |  |  |  |
| Outpatient visits | 0.0% | 0.2% | 0.0% | 0.0% | 0.1% | 0.0% | 0.2% | 0.0% |
| Road traffic accidents | 0.0% | 0.0% | 0.2% | 0.0% |  |  |  |  |
| Deaths |  |  |  |  |  |  |  |  |
| Emergency room deaths |  |  |  |  |  |  |  |  |
| ICU deaths | 0.0% | 0.0% | 0.0% | 0.0% |  |  |  |  |
| In patient deaths | 1.4% | 0.0% | 0.1% | 0.0% | 0.1% | 0.3% | 0.1% | 0.3% |
| Maternal deaths | 0.0% | 0.0% | 0.0% | 0.0% | 0.0% | 0.0% | 0.0% | 0.0% |
| Newborn deaths | 0.0% | 0.0% | 0.0% | 0.0% | 0.0% | 0.1% | 0.0% | 0.0% |
| Stillbirths | 0.0% | 0.0% | 0.0% | 0.0% | 0.1% | 0.1% | 0.0% | 0.1% |
| Trauma deaths | 0.0% | 0.0% | 0.1% | 0.0% |  |  |  |  |

**Supplementary Table 4. Positive and negative outliers in the pre-pandemic and pandemic periods (Lao PDR)**

|  | Lao PDR | | | |
| --- | --- | --- | --- | --- |
|  | Positive pre | Negative pre | Positive pandemic | Negative pandemic |
| RMNH services |  |  |  |  |
| Family planning | 0.2% | 0.1% | 0.2% | 0.0% |
| Antenatal care services | 0.4% | 0.0% | 0.1% | 0.0% |
| Deliveries | 0.1% | 0.0% | 0.1% | 0.0% |
| Cesarean section | 0.1% | 0.5% | 0.0% | 0.6% |
| Neonatal resuscitation |  |  |  |  |
| Kangaroo mother care |  |  |  |  |
| Postnatal care visits | 0.2% | 0.0% | 0.1% | 0.0% |
| Child health services |  |  |  |  |
| Acute malnutrition visit | 0.1% | 0.1% | 0.2% | 0.2% |
| Diarrhea visit | 0.1% | 0.0% | 0.3% | 0.0% |
| Pnuemonia visit | 0.0% | 0.0% | 0.4% | 0.0% |
| BCG vaccines | 0.1% | 0.0% | 0.1% | 0.0% |
| Measles vaccine | 1.1% | 0.0% | 0.0% | 0.1% |
| Polio vaccine | 0.0% | 0.1% | 0.0% | 0.0% |
| Pentavalent vaccine | 0.0% | 0.1% | 0.0% | 0.0% |
| Pneumococcal vaccine | 0.7% | 0.0% | 0.0% | 0.0% |
| Rotavirus vaccine |  |  |  |  |
| Fully vaccinated 1 year old |  |  |  |  |
| Infectious disease services |  |  |  |  |
| HIV testing | 0.2% | 0.1% | 0.1% | 0.2% |
| ART |  |  |  |  |
| HIV suppression |  |  |  |  |
| TB screening |  |  |  |  |
| TB treatment |  |  |  |  |
| NCD services |  |  |  |  |
| Cervical cancer screening |  |  |  |  |
| Diabetes screening |  |  |  |  |
| Diabetes treatment | 0.3% | 0.1% | 0.2% | 0.1% |
| Hypertension screening |  |  |  |  |
| Hypertension treatment | 0.3% | 0.0% | 0.2% | 0.0% |
| Other services |  |  |  |  |
| Emergency room visits | 0.7% | 0.0% | 0.4% | 0.0% |
| In patient visits | 0.0% | 0.7% | 0.4% | 0.3% |
| Trauma visits |  |  |  |  |
| ICU visits |  |  |  |  |
| Outpatient visits | 0.1% | 0.0% | 0.2% | 0.0% |
| Road traffic accidents |  |  |  |  |
| Deaths |  |  |  |  |
| Emergency room deaths |  |  |  |  |
| ICU deaths |  |  |  |  |
| In patient deaths | 0.1% | 0.3% | 0.1% | 0.3% |
| Maternal deaths | 0.0% | 0.0% | 0.0% | 0.0% |
| Newborn deaths | 0.0% | 0.1% | 0.0% | 0.0% |
| Stillbirths | 0.1% | 0.1% | 0.0% | 0.1% |
| Trauma deaths |  |  |  |  |
